# *JAHA*: The American Heart Association's Open Access Journal

**DOI:** 10.1161/JAHA.111.000638

**Published:** 2012-02-20

**Authors:** Joseph A Vita

**Affiliations:** Journal of the American Heart AssociationBoston, MA

I am pleased to introduce the *Journal of the American Heart Association (JAHA)*, the American Heart Association's new Open Access journal. The Scientific Publishing Committee created *JAHA* to align the American Heart Association with recent trends in the publishing industry as readers have moved away from the print version of journals and increasingly seek rapid electronic access to scientific articles. In addition, *JAHA* was conceived as a novel publication forum to represent the collective interests of the 16 Scientific Councils (Table 1).

**Table 1.** Scientific Councils of the American Heart Association

Council on Arteriosclerosis, Thrombosis and Vascular Biology (ATVB)
Council on Basic Cardiovascular Sciences (BCVS)
Cardiopulmonary, Critical Care, Perioperative and Resuscitation (3CPR)
Council on Cardiovascular Surgery and Anesthesia (CVSA)
Council on Cardiovascular Disease in the Young (CVDY)
Council on Cardiovascular Nursing (CVN)
Council on Cardiovascular Radiology and Intervention (CVRI)
Council on Clinical Cardiology (CLCD)
Council on Epidemiology and Prevention (EPI)
Council on Functional Genomics and Translational Biology (FGTB)
Council for High Blood Pressure Research (HBPR)
Council on the Kidney in Cardiovascular Disease (KCVD)
Council on Nutrition, Physical Activity and Metabolism (NPAM)
Council on Peripheral Vascular Disease (PVD)
Council on Quality of Care and Outcomes Research (QCOR)
Stroke Council (STROKE)

*JAHA* publishes original articles, editorials, and reviews of cardiovascular and cerebrovascular science in an online-only format. Articles undergo rigorous peer review by a group of editors, statistical consultants, and reviewers representing the full breath and strength of the American Heart Association. Because articles are published exclusively in electronic form, we have no need to place limits on article length or the number of references. Authors have unrestricted use of color images and video to present all aspects of their work. Furthermore, the online-only format allows us to prepare articles for publication more rapidly than print journals. The editorial team will strive to process submissions and post accepted papers on our web page and in PubMed Central as quickly as possible, while maintaining the integrity of the peer review process.

Most importantly, readers of *JAHA* have unrestricted subscription-free access to all journal content from any device connected to the Internet. In contrast to the other American Heart Association journals, *JAHA* does not depend on subscription revenue. Publication costs are borne by the author, their institution, and/or the sponsor of the work, with a substantial discount for Professional Members of the American Heart Association. The open access publishing model has many advocates because it facilitates the dissemination of scientific knowledge. Available evidence suggests that the open access format leads to more downloads and may increase citations compared with articles published in the non–open access format, possibly reflecting wider dissemination and global access to the work.^1–3^

Since announcing the launch of *JAHA* at Scientific Sessions in November 2011, the editors have often been asked about the types of articles we are going to publish. We envision *JAHA* as a forum for high-quality original articles that cover the full range of cardiovascular science, including basic science, translational science, clinical trials, and epidemiological and outcomes research. In keeping with this idea, we have established formal links with the 16 Scientific Councils. Many of the Councils represent disciplines that previously lacked a publication venue within the American Heart Association. We have included a representative of each Scientific Council on our Editorial Board, who will review and, at times, serve as Associate Editor for papers in their discipline. In this latter capacity, the board members will select reviewers and suggest the most appropriate editorialists who can put the work into the proper perspective for our readers. We hope that authors in any domain of cardiovascular research will consider *JAHA* when they want their work to have rapid review and the widest possible dissemination.

Another common question is how *JAHA* will relate to the other American Heart Association journals. We are currently in the process of establishing formal links with the editors of *Arteriosclerosis*, *Thrombosis*, *and Vascular Biology*, *Circulation Research*, *Hypertension*, *Stroke*, and the *Circulation* family of journals. The editors of these journals often have had to reject high-quality papers to avoid exceeding their printed page budgets. The editors will communicate with us about such papers, and when appropriate, we will invite the authors to submit to *JAHA*. Authors will be asked to respond to the original reviews, and the *JAHA* editors will consider the paper without necessarily sending the paper out to new reviewers.

Our launch includes a number of outstanding papers that exemplify our goal to publish a broad range of cardiovascular science. A translational study by Ammirati et al links a specific T-lymphocyte subset to the extent of atherosclerosis in human subjects and a murine dyslipidemia model.^4^ An editorial by Dr Paul Ridker puts these findings into context with recent developments in the field of inflammation and antiinflammatory therapy for the treatment of cardiovascular disease.^5^ We also are publishing the results of a well-conducted clinical trial by Singer et al comparing the effects of antiplatelet therapies on claudication in patients with peripheral arterial disease (PAD).^6^ This paper is important because of the need for more comparative effectiveness research to guide the management of peripheral arterial disease and the importance of publishing both positive and negative studies. Fonarow et al showed that the National Institutes of Health Stroke Scale (NIHSS) provides strong prognostic information about 30-day mortality risk in Medicare beneficiaries with acute ischemic stroke.^7^ The forthcoming editorial by Dr Edward Jauch, Chair of the American Heart Association Stroke Council, explains how these findings may be used to better evaluate the performance of centers that manage patients with ischemic stroke.^8^ Finally, Barkoudah et al provide an interesting analysis of published clinical trials in patients with Type 2 diabetes mellitus providing new information about the increasingly recognized links between renal disease and cardiovascular mortality.^9^

Our first articles and editorials demonstrate our commitment to publish a wide range of cardiovascular science in *JAHA*. The editors believe that the open access format represents the future of scientific publishing and we are excited and honored to oversee the American Heart Association's first open access journal. We look forward to working with our authors to speed the dissemination of high-quality science to our readers with the ultimate goal of increasing our understanding of the underlying mechanisms and improving the management of patients with cardiovascular disease and stroke.
